# Changes in the proteomic profile of athletes’ plasma associated with exercise intensity

**DOI:** 10.1038/s41598-026-44729-5

**Published:** 2026-03-19

**Authors:** Kristina A. Malsagova, Tatiana V. Butkova, Kirill S. Nikolsky, Denis V. Petrovskiy, Arthur T. Kopylov, Valeriya I. Nakhod, Liudmila I. Kulikova, Vladimir R. Rudnev, Ksenia A. Yurku, Evgenii I. Balakin, Anastasiia S. Bukreeva, Alexander A. Izotov, Vasiliy I. Pustovoyt, Anna L. Kaysheva

**Affiliations:** 1 Institute of Biomedical Chemistry, Moscow, 119121 Russian Federation; 2 State Research Center-Burnasyan Federal Medical Biophysical Center of Federal Medical Biological Agency, Moscow, 123098 Russian Federation

**Keywords:** Sport, Circulating proteins, Proteome, De novo peptide sequencing, DIA-MS, Biochemistry, Biological techniques, Biomarkers, Computational biology and bioinformatics

## Abstract

High physical activity often results in complex and ambiguous proteomic changes. This study aimed to analyze possible changes in the composition and content of proteins in plasma samples of elite athletes from sports with various activity levels and intensity. We performed proteomic profiling of blood plasma from 93 elite athletes with over eight years of continuous professional experience. High-resolution tandem mass spectra obtained in the data-independent acquisition mode were analyzed using the PLGS algorithm against a library of known protein sequences, and the PowerNovo algorithm for de novo protein sequencing. Combining the two protein identification strategies improved the completeness of the analysis and expanded the number of identified proteins. We identified changes in levels of circulating proteins that distinguish the high-intensity group from other groups by proteins involved in immune response, lipid transport and metabolism, and oxygen and iron transport. Changes in protein levels in biological samples of professional athletes may be associated with the training load intensity and type of sport.

**Supplementary Information:**

The online version contains supplementary material available at 10.1038/s41598-026-44729-5.

## Introduction

An athlete’s molecular profile, characterized using omics technologies, reflects the composition and content of proteins, metabolites, and other endogenous biomolecules in a biological sample^1^. Analyzing such profiles is of importance for understanding the impact of physical activity and loading. Information on changes in metabolites and proteins may shed light on the modulation of biological processes in response to training and competition, aiding in health monitoring and optimization of training^2–4^.

Emerging omics studies in sports medicine explore potential applications, such as oxidative stress management, immune response regulation, and hydration assessment^5,6^. Ongoing research aims to develop personalized medicine approaches that adapt training and recovery to individual biological characteristics^7^.

**Fig. 1:**
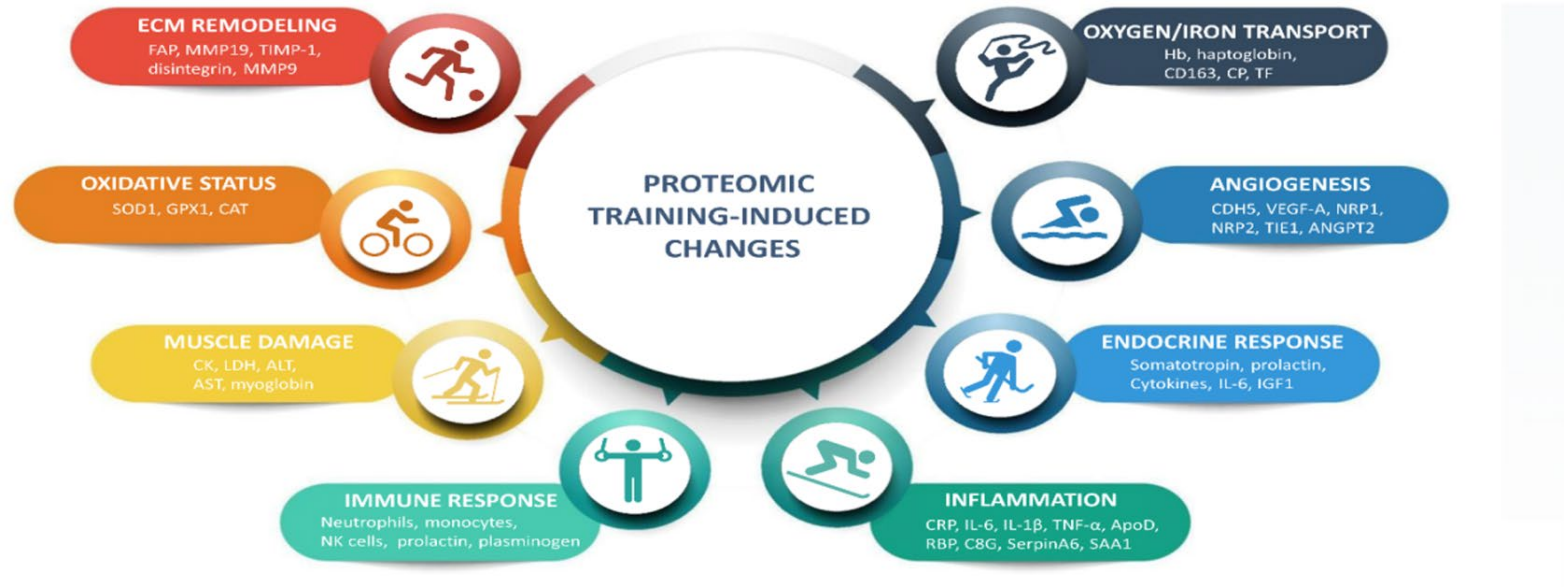
Proteomic changes in plasma in response to physical activity. Several biological processes and corresponding proteins found in the study are discussed in the context of long-term physical activity in professional athletes. Hb – hemoglobin, CD136 – macrophage-stimulating protein receptor, CP – ceruloplasmin, TF – transferrin, CDH5 – cadherin-5, VEGF-A – vascular endothelial growth factor A, NRP1/2 – neuropilin 1/2, TIE1 – tyrosine-protein kinase receptor, ANGPT2 – angiopoietin-2, ILs – interleukins, IGF1 – insulin-like growth factor 1, CRP – C-reactive protein, TNF – tumor necrosis factor, ApoD – apolipoprotein D, RBP – plasma retinol binding protein, C8G – complement component C8 gamma, SAA1 – serum amyloid A-1 protein, NK cells – natural killer cells, CK – creatine kinase, LDH – lactate dehydrogenase, ALT – alanine aminotransferase, AST – aspartate aminotransferase, SOD1 – superoxide dismutase 1, GPX1 – glutathione peroxidase 1, CAT – catalase, FAP – prolyl endopeptidase FAP, MMP9/19 – matrix metalloproteinase 9/19, TIMP1 – metalloproteinase inhibitor 1.

Regular, prolonged physical activity alters the level of circulating blood proteins involved in angiogenesis, homeostasis maintenance, oxygen transport, oxidative stress, inflammation, and immune response^8,9^. These changes are likely associated with adaptation in cardiorespiratory endurance^9^, immune homeostasis maintenance^10–12^, hormonal^13^ and neuronal changes, metabolic changes^14,15^, oxidative stress^16^, and inflammation^17^ (Fig. 1).

Blood plasma represents the most complex proteome, encompassing tissue proteomes as multiple subsets^18^. It is the most frequently studied proteome in both clinical and scientific research settings, because plasma proteins play a vital role in the regulation of nearly all physiological processes.

This study performed proteomic profiling of 93 plasma samples obtained from three groups of elite athletes based on sports intensities. High-resolution tandem mass spectra from data-independent acquisition mode were analyzed using the PLGS algorithm against a library of known protein sequences, and the PowerNovo algorithm for *de novo* protein sequencing. Making a “digital portrait” of an athlete’s protein composition is promising for sports medicine, reflecting modulated biological processes at the molecular level.

Currently, only a few publicly available studies have examined features of plasma proteins composition in relation to exercise intensity and sport type. Such features remain insufficiently studied, despite their important role for the understanding of adaptive mechanisms to regular and intense physical exercise.

## Materials and methods

### Subjects

A total of 93 athletes were enrolled and their basic anthropometric parameters are shown in Table 1. Athletes were divided into groups based on the classification by J. Mitchell et al., which categorizes sports by the type and intensity of physical activity and associated cardiovascular demands^19^. Participants were assigned according to the peak dynamic load characteristic of their sport, defined as the percentage of maximal oxygen uptake (MaxO₂) achieved. All participants trained systematically under coach supervision. Athletes were in a general preparatory period characterized by relatively high volume, reduced intensity of main exercises, and diverse training methods. At the time of the study, athletes underwent an in-depth medical examination assessing health status, adaptation to load, and detection of latent pathologies (cardiovascular, endocrine, etc.). The examination included a 24-parameter assessment (medical/sports history, training load, recovery, performance) and an extended 56-parameter blood analysis (biochemical, lipid, hormonal, immunological profiles).

**Table 1:** Anthropometric, clinical, and psychometric characteristics of the participants.

Characteristic	Group “high”		Group “moderate”		Group “low”	
Participants	Men (*n* = 21)	Women (*n* = 16)	Men (*n* = 42)	Women (*n* = 1)	Men (*n* = 11)	Women (*n* = 2)
Kind of sport	Beach Soccer, Biathlon, Football, Kayaking and canoeing, Rowing, Ski race	Biathlon, Kayaking and canoeing, Rowing, Ski race, Biathlon	Athletics, Figure skating, Freestyle wrestling, Greco-Roman wrestling	Athletics	Sailing, Sambo	Sailing
Age, years	25 ± 3	21 ± 3	29 ± 3	20 ± 0	30 ± 6	24 ± 1
Length of time engaged in sports, years	12 ± 4	12 ± 5	20 ± 4	11 ± 0	21 ± 7	16 ± 6
BMI, kg/m^2^*	24 ± 1	23 ± 2	25 ± 5	–	24 ± 2	20 ± 1
Maximum heart rate, beats per minute	139 ± 16.6	148 ± 19	140 ± 14.5	–	136 ± 13	159 ± 0.7
Chronic diseases	No chronic or serious illnesses, no heredity burden					
Epidemiological history (last 6 months)	Epidemiological history is negative					
Therapeutic use exemption for a prohibited substance or method	Absence					
Allergies and food intolerances	Allergic rhinitis to animal hair (*n* = 1); hay fever (*n* = 1); contact dermatitis (*n* = 1)	Hay fever (*n* = 1); drug allergy (*n* = 1)	Food intolerance (*n* = 1); hay fever (*n* = 1)	Absence	Hay fever (*n* = 2); allergic rhinitis to animal hair (*n* = 1)	Absence
Dynamics of sports results for the year	Improvement (*n* = 11); Stable (*n* = 10)	Improvement (*n* = 8); Stable (*n* = 8)	Improvement (*n* = 9); Stable (*n* = 33)	Stable (*n* = 1)	Improvement (*n* = 4); Stable (*n* = 7)	Improvement (*n* = 1); Stable (*n* = 1)
Number of sports training sessions per day	2–3		2		2	
Number of rest days per week	1–2		1		1	
Bad habits	Absence					
Inclusion criteria	Male and female professional athletes with at least 5 years of training experience residence in the central region of the Russian Federation					
Exclusion criteria	Age under 18 presence of acute or chronic diseases affecting metabolism or physical activity recent injuries or surgical interventions (less than 6 months before participation) use of anabolic steroids, hormonal drugs, or other prohibited substances pregnancy or lactation (for female participants) refusal to sign the informed consent form					

*According to available data; BMI – body mass index (kg/m^2^).

The study involved athletes from three groups with varying training load intensities:

1. “High” group: Average age 25 ± 3 years (men) and 21 ± 3 years (women); 12 years average experience; BMI 24 ± 1 kg/m² (men), 23 ± 2 kg/m² (women).
2. “Moderate” group: Average age 29 ± 3 years (men), 20 ± 0 years (women); 20 ± 4 years experience (men), 11 ± 0 years (women); BMI 25 ± 5 kg/m².
3. “Low” group: Average age 30 ± 6 years (men), 24 ± 1 years (women); 21 ± 7 years experience (men), 16 ± 6 years (women); BMI 24 ± 2 kg/m² (men), 20 ± 1 kg/m² (women).

The maximum heart rate (HR) of the study participants varied depending on the group and gender. In the “High” group, the average maximum HR was 139 ± 16.6 bpm for men and 148 ± 19 bpm for women. These values reflect a high level of physiological adaptation to intense physical activity and may indicate good cardiovascular fitness in athletes with a high degree of sports involvement.

In the “Moderate” group, the average maximum HR in men was similar to that in the “High” group, at 140 ± 14.5 bpm. However, no data were available for women in this category, which prevented a gender comparison within the moderate intensity group.

In the “Low” group, a slightly different pattern was observed. The average maximum HR in men was 136 ± 13 bpm. Interestingly, the maximum HR in women with this lower level of physical activity was the highest, at 159 ± 0.7 bpm.

Participants had no chronic diseases, negative family history, negative epidemiological history for the past six months, and no therapeutic use exemptions for prohibited substances. Allergies and intolerances were recorded as per Table 1. Sports performance dynamics over the past year showed predominantly positive or stable results across groups.

Analysis of allergies and food intolerance showed that in the “High” group, among men, there were cases of allergic rhinitis to animal hair (*n* = 1), hay fever (*n* = 1), and contact dermatitis (*n* = 1); among women, there were cases of hay fever (*n* = 1) and drug allergy (*n* = 1). In the “Moderate” group, among men, there were cases of food intolerance (*n* = 1) and hay fever (*n* = 1); among women, no allergic reactions were reported. In the “Low” group, among men, there were cases of hay fever (*n* = 2) and allergic rhinitis to animal hair (*n* = 1); among women in this group, no allergic diseases were detected.

The dynamics of sports results were analyzed over the last year before the start of the study. In the “High” group, positive dynamics prevailed among both men and women: among men, an improvement in sports performance was recorded in 11 people, and a stable level in 10 people; among women, an improvement in 8 people and a stable result in 8. In the “Moderate” group, an improvement was noted among 9 male athletes, and a stable result in 33; among women, the dynamics remained stable (*n* = 1). In the “Low” group, an improvement in sports results was recorded among 4 men and a stable level in 7; among women, there were cases of both improvement and stable results.

This pilot study exhibits a gender and numerical imbalance between groups, particularly the insufficient sample sizes for the female subgroups in the “Moderate” and “Low” categories for robust statistical analysis (Table 1). To mitigate this effect, comparative analyses were conducted separately for males and females, with formal statistical analysis performed solely on the male cohorts. The limited statistical power for the female subgroups and the attendant limitations on interpretation are addressed in the “Limitations” section.

The control group in the present study was based on data we obtained earlier in 2022 for apparently healthy participants, which are presented in the ProteomeXchange Consortium database^20^ via the PRIDE partner repository with the dataset identifier PXD035863 (https://www.ebi.ac.uk/pride/archive/projects/PXD035863). The control group consisted of samples from ten men and ten women age-matched to the athletes (men 25.5 ± 4 years old, women 24 ± 3.2 years old) were students and employees, leading a sedentary lifestyle involving prolonged sitting. Their BMI was within the normal range. Proteomic data from this group were used as an additional reference for potential protein content in the plasma of sedentary individuals. According to the survey, six men and seven women were non-smokers, while the rest were long-term smokers. The body mass index (BMI) of the participants was within the normal range: 24.1 ± 3.5 kg/m^2^ in men and 22.1 ± 3.5 kg/m^2^ in women. Based on the criterion of the presence of somatic diseases during their lifetime, five men and eight women had no chronic or severe diseases. The men had non-drug remission of bronchial asthma (*n* = 1), hypertension (*n* = 1), remission of chronic tonsillitis (*n* = 1), remission of gastroduodenitis (*n* = 1), and varicocele (*n* = 1). In women, there was pyelonephritis in remission (*n* = 1) and ovarian cysts (*n* = 1). The proteomic data from the control group served as a supporting dataset reflecting the proteomic features of sedentary people. These data were not used in statistical tests and the information is for reference purposes only.

### Informed consent

The study was approved by the Ethics Committee of the A.I. Burnazyan State Research Center, FMBA of Russia (Protocol no. 40, 18.11.2020). Informed consent was obtained from all participants after a comprehensive explanation of objectives and procedures. Confidentiality and data protection were assured. The study complied with the Declaration of Helsinki.

### Analysis

Participants were advised to avoid high-intensity exercise, stimulating foods and drinks, alcoholic beverages, and supplements on the day before blood collection. Following overnight fasting, blood samples were collected between 8 and 10 a.m. in 3.8% sodium citrate tubes (Improvacuter, Guangzhou Improve Medical Instruments Co., Ltd., Guangzhou, China). Plasma was separated by centrifugation at 3,000 rpm for 6 min at room temperature, aliquoted into 500 µL, and stored at -80 °C.

Protein extraction and digestion followed a standard protocol involving reduction, alkylation, and tryptic digestion. Briefly, 2 µL of plasma was added to 8 µL of denaturation buffer (5 M urea, 1% sodium deoxycholate solution, 300 mM sodium chloride, 10% acetonitrile solution, and 100 mM triethylammonium bicarbonate (TEAB, pH 8.2–8.5)) supplemented with 10 mM TCEP. The sample was incubated at 40 °C for 20 min with stirring at 800 rpm. Then 2 µL of 1% 4-vinyl pyridine in isopropanol was added and the resulting sample was incubated at room temperature in darkness for the next 20 min. The sample was diluted to 100 µL with 60 mM TEAB to reduce the concentration of urea and sodium deoxycholate solution, and an aliquot of 200 ng of trypsin was added twice every 3 h for digestion at 37 °C. The reaction was stopped by acidification (10 µL of 10% formic acid), and the sample was centrifuged at 12,000 *g* for 10 min at room temperature to sediment deoxycholic acid. The resulting supernatant was dried under vacuum at 45 °C and resuspended in 20 µL of 0.1% formic acid^21^.

The analysis was performed on a quadrupole time-of-flight mass spectrometer Xevo™ G2-XS Q-tof (Waters, Wilmslow, UK) equipped with an Acquity™ UPLC H Class Plus chromatography system (Waters, Wilmslow, UK). Precursor ions were surveyed in the hybrid information-independent (DIA) MS^E^-SONAR mode. Peptides were separated in a gradient of mobile phase A: 0.1% formic acid in water; B: 0.1% formic acid in acetonitrile. Chromatographic separation used a 53-min gradient for B: 1.5 min 3%, 26.5 min 19%, 42 min 32%, 43.5 min 97%, 47.5 min 97%, 49 min 3%, 52 min 3% (flow 0.2 ml/min, except 0.3 ml/min at 47.5 min). Data are available on Nikolsky, Kirill (2024), “Proteomic Analyses of Blood Samples of Highly Trained Athletes”, Mendeley Data, V2, doi: 10.17632/p8ts76xmfd.2.

Proteomic data were processed with PLGS software (v3.0.3) using the UniProt KB database. Identification was performed settings with the following parameters: precursor mass tolerance 50 ppm, fragment ion tolerance 100 ppm, the identified sequence is at least 10 amino acids long, trypsin enzyme, up to two missed cleavages, FDR < 5% (set at 1%). Fixed modifications: “S-pyridylethyl + C”, “Deamidation + N”. Variable modifications: “Deamidation + Q”, “Oxidation + M”.

De novo analysis used the PowerNovo tool^22^ processed with the following key parameters: contigs constructed using the ALPS de Bruijn assembler (k-mer = 8); mapped contigs aligned to reference sequences using a BLAST-like algorithm, requiring at least 75% identity^22^. All peptides identified by de novo services were verified using quality control criteria: only peptides with a length ≥ 5 amino acid residues, the identified sequence is at least 10 amino acids long, precursor mass tolerance of 50 ppm, fragment ion tolerance 100 ppm, and a PowerNovo score ≥ 0.75 were accepted. For reproducibility, proteins were deemed reliable only if detected in at least five samples.

Comparative analysis used Python/Pandas scripts data analysis library (https://pandas.pydata.org/). Peptide sequences from both strategies were normalized and clustered with more than 95% similarity as well as matched sequences of different lengths (de novo contigs, the missed trypsin cleavage site) using Usearch engine (https://www.drive5.com/usearch/)).

Results were visualized with Plotly library (https://plotly.com/python/). Protein information was enriched using an in-house custom library (available at GitHub: https://github.com/protdb/uniprot_meta_tool).

Concentrations of proteins were calculated based on label-free quantification results. For athlete groups, the following formula was used:

$${C}_{prot}=\frac{{F}_{prot}}{100}\times{C}_{alb}$$

where *F*_*prot*_ is a value based on top 3 peptide intensities (as % of albumin, calculated by PLGS), and *C*_*alb*_ is albumin concentration 40 g/L.

Absolute concentrations in the control group were calculated using the following formula:

$${C_i}={C_{total}}=\frac{{emPA{I_i} \times {M_i}}}{{\sum\nolimits_{{i=1}}^{N} {(emPA{I_i} \times {M_i})} }}$$

where C_*total*_ – is the total plasma protein concentration 75 g/L, *emPAI*_*i*_ is the exponentially modified protein abundance index value for the protein (calculated by PeptideShaker on MaxQuant data), and *M*_*i*_ is the molecular weight of the protein.

The reference values of plasma concentrations of proteins for Fig. 3 and Supplementary were taken from Protein Atlas API^23^.

The P-value was determined by Mann-Whitney U rank test^24^ in the scipy.stats package^25^. False discovery control (FDC) was performed with the Benjamini-Hochberg method^26^ also using the scipy.stats package^27^. FDC was applied across all pairwise and one-vs-rest comparisons for each protein (each group against every other group; each group against all remaining groups combined) as a single family of hypotheses, while each protein was processed with FDC independently. The same approach including programming packages, statistical tests (Mann-Whitney U test with Benjamini-Hochberg correction) and FDC application logic were employed to analyze and visualize clinical data (Fig. 5).

The study was performed using the equipment of the Human Proteome Core Facilities at the Institute of Biomedical Chemistry (Russia).

Functional parameters were measured during the cycle ergometer test, which was conducted at a temperature of 22 °C and 60% relative humidity. During testing, participants wore sports shorts, socks, and shoes. Throughout the examination, heart rate (HR) and ECG parameters were continuously recorded using a Cosmed “Quark C12” (Italy) wired recorder to assess cardiac electrical activity. Pulmonary ventilation and metabolic response parameters were continuously measured using Cosmed “Quark CPET” systems (Italy).

Anthropometric characteristics, including height, weight, and BMI, were measured once during a comprehensive medical examination.

Biochemical and immunochemical analyses were performed on a Cobas 6000 modular platform (Roche Diagnostics, Germany) according to the manufacturer’s instructions^28^. A total of 56 parameters (biochemical, lipid, hormonal, and immunological profiles) were assessed, including lactate, total acid phosphatase, triglycerides, CROSSL (C-terminal telopeptides of type I collagen), and creatine kinase.

## Results

### De novo enriches the results of protein sequence database search analysis

**Fig. 2:**
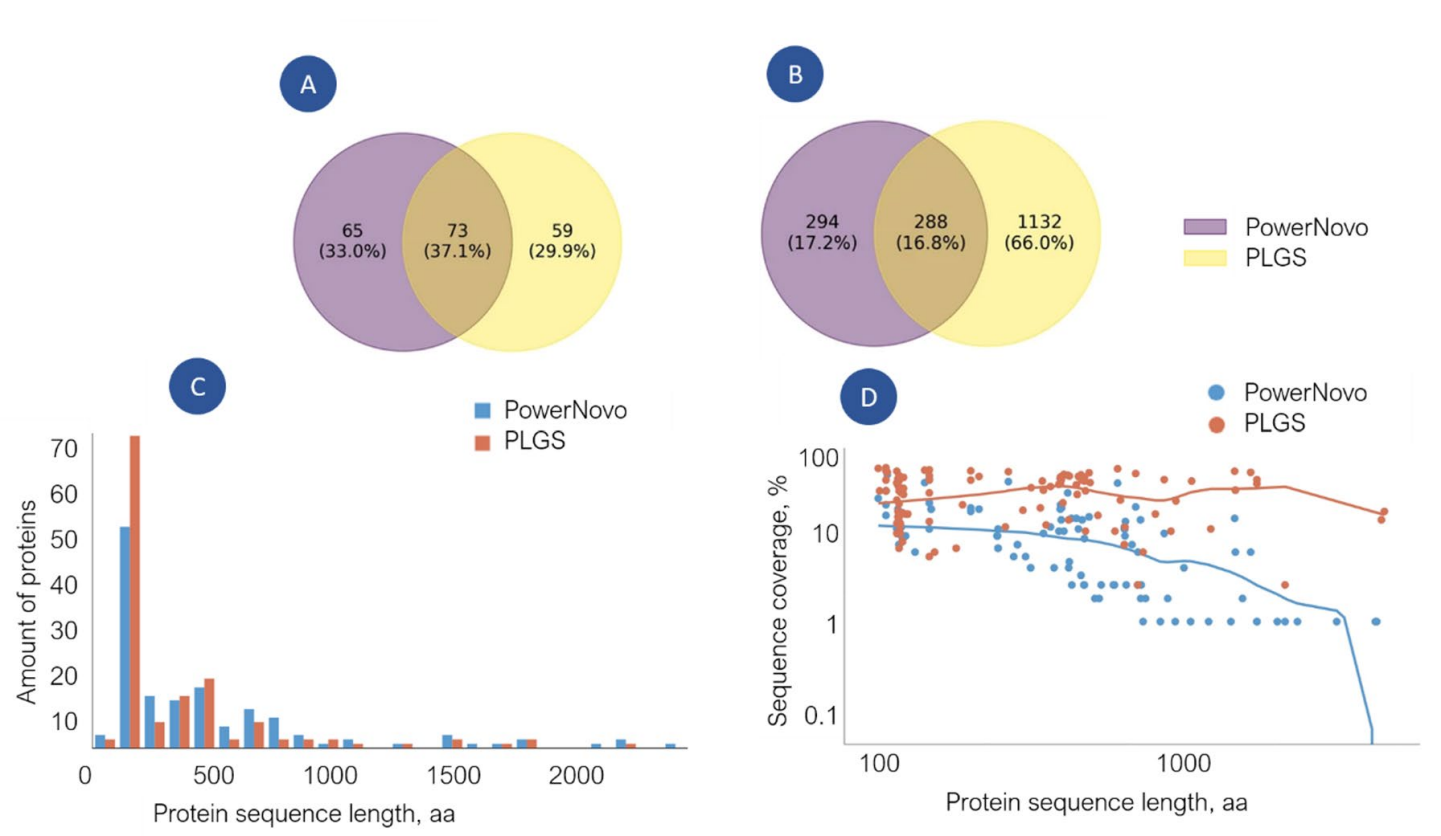
Matching identifications between the two strategies PowerNovo and PLGS. Identification conditions: peptide length at least 5 a.a. residues, peptide and fragment ions tolerance is 50 and 100 ppm, respectively, detected in at least five distinct plasma samples in the comparison groups, FDR < 1% (PLGS), score ≥ 0.75 (PowerNovo). Venn diagrams plotting the number of reported proteins for each inference algorithm (PowerNovo is violet colour; PLGS is yellow colour): (**a**) the number of identified proteins; (**b**) the number of identified peptides; (**c**) distribution histogram of the protein identification number depending on the sequence length for each search strategy. The histogram illustrates the distribution of identified proteins (Y-axis: “Amount of proteins”) depending on the length of their amino acid sequence (X-axis: “Protein sequence length, aa”). Data for PowerNovo (red) and PLGS (blue) are highlighted in the graph; (**d**) the scatter plot of protein amino acid sequence coverage by identifications, depending on the protein length, for each search strategy. The diagram shows the relationship between protein length (X-axis: “Protein sequence length, aa”) and the percentage of its amino acid sequence coverage by identified peptides (Y-axis: “Sequence coverage, %”). Each point corresponds to an individual protein identified by one of the strategies (indicated by different colours). The blue and red smoothing lines are obtained using the LOWESS (Locally Weighted Scatterplot Smoothing) method, which constructs a flexible curve reflecting local data trends without assuming linearity. The graph allows us to assess the completeness of identification (coverage) depending on protein size and compare the effectiveness of the two strategies in this aspect. Data for PowerNovo (red) and PLGS (blue) are highlighted in the graph.

The number of identified proteins is determined by the sample preparation stage: the use of fractionation and depletion (removal of highly abundant proteins), which reduces sample complexity and deepens proteomic coverage; the parameters of chromatographic separation (flow rate and gradient duration); and the conditions of post-analytical data processing like the permissible mass measurement accuracy (tolerance) and the requirements for the frequency of identifications in the sample set. In this study, the combination of these factors entails a moderate number of identifications. We did not use sample fractionation but employed micro-flow chromatography instead, and established stringent verification criteria: 50 and 100 ppm for the precursor ions and fragment ions, respectively, the identified sequence is at least 10 amino acids long, and required identifications to be present in at least five samples. A total of 197 proteins were identified (Fig. 2a), among which PLGS identified 132 proteins, while PowerNovo detected 138. For peptides, PLGS identified 1714 sequences and PowerNovo 582, with 288 common to both (Fig. 2b), thus the use of these tools complimentarily increases the number of protein and peptide identifications. The obtained data are the expected result at the chosen experimental design, and the depth and reliability of the identifications remain at a level comparable to other DIA-MS studies (see details in the Supplementary Materials, Figures S1 and S2).

The distribution of number of the protein identification depends on the protein length (Fig. 2c). More proteins were identified in the 100–1000 amino acid range, with PowerNovo identifying significantly more than PLGS across almost the entire length range. However, PLGS provided greater amino acid sequence coverage, especially for medium and large proteins with ≥ 1000 aa (Fig. 2d). Comparison of the two identification strategies exposed the advantage of PLGS over PowerNovo: both strategies returned approximately the same efficiency of sequence identification coverage for small proteins (about 300 amino acid residues) whereas for medium and large-sized proteins (≥ 500 aa) PLGS is more efficient.

**Fig. 3:**
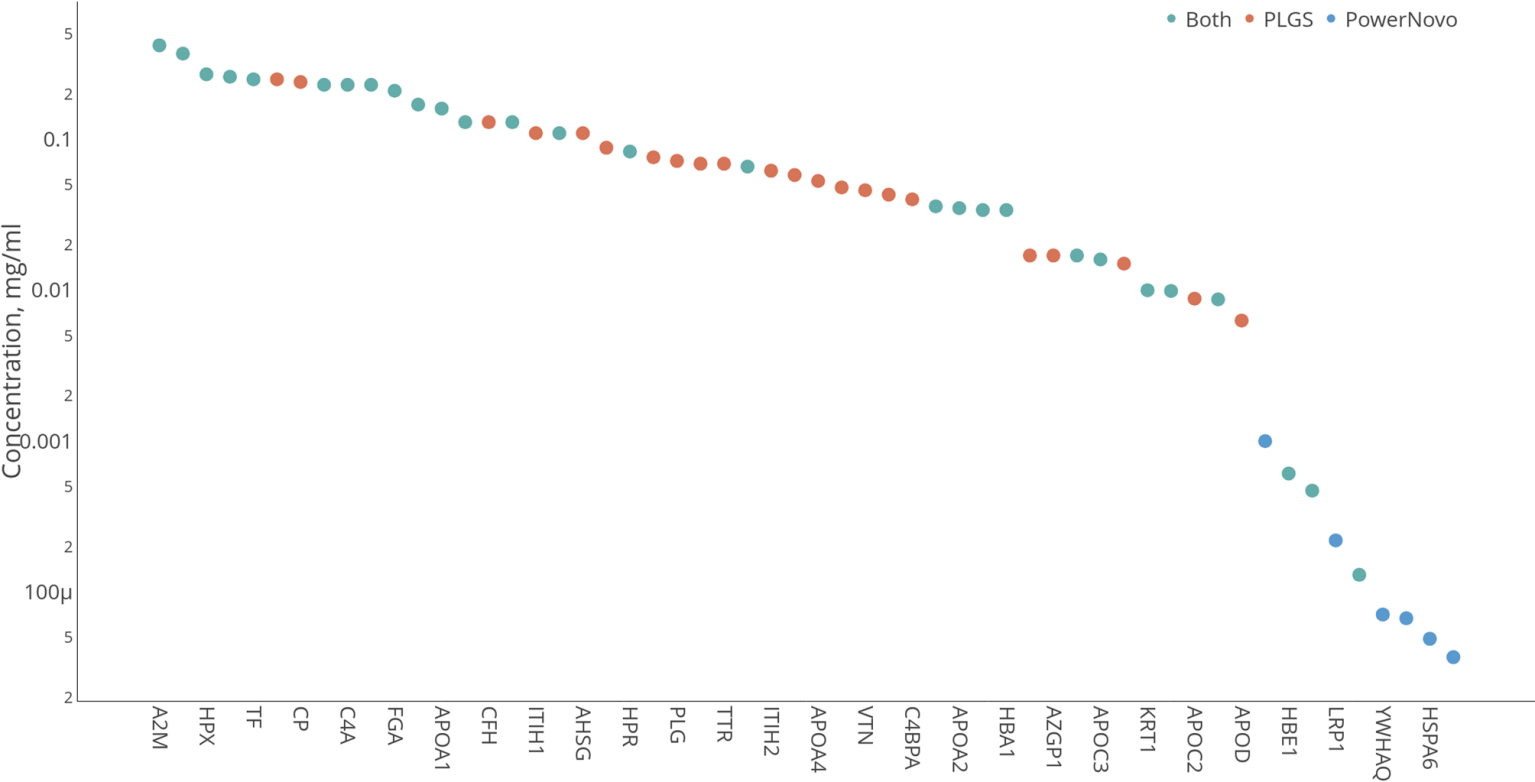
Concentrations of identified circulating proteins depicted as the abundance levels of proteins identified by at least one of the two search strategies (PLGS or PowerNovo), or common to both approaches. Proteins are ranked and grouped by their concentration ranges. The X-axis shows the genes of the identified proteins, the Y-axis “Concentration, mg/mL” shows the protein concentration ranges. The axis is on a logarithmic scale to cover the wide dynamic range of concentrations. Grouping of proteins by abundance: the left part of the graph corresponds to high-copy number proteins (> 1 µg/mL); proteins grouped in the center correspond to the range of moderate-copy number proteins (1 µg/mL to 10 ng/mL); proteins shown on the right correspond to the range of low-copy number proteins (< 10 ng/mL). Proteins identified by both strategies (Common) are shown in green; proteins identified by PLGS are shown in red; proteins identified by PowerNovo are shown in blue. The figure demonstrates that most high-abundance proteins are identified by both methods (green markers), which highlights the complementarity of the approaches. As the concentration decreases, unique identifications specific to each strategy appear (red and blue markers). In the low-concentration region, identifications from PowerNovo are more frequent.

Analysis of identification “depth” revealed a high matching level. Figure 3 shows proteins identified in athlete plasma samples categorized by abundance (the Protein Atlas data^23^: high (> 1 µg/mL), moderate (1 µg/mL–10 ng/mL), and low (< 10 ng/mL). In the high-abundance zone, 49 identifications were found, mostly by both strategies. These included proteins involved in transport of biomolecules, in particular iron (GO:0006826, *n* = 4, FDR = 0.00087), oxygen and carbon dioxide (GO:0015670, *n* = 3, FDR = 0.00070), cholesterol (GO:0030301, *n* = 9, FDR = 1.01 × 10^–10^), heme, etc., as well as transport of supramolecular complexes, including regulation of vesicle-mediated transport (GO:0060627, *n* = 16, FDR = 1.36 × 10–10); proteins maintaining homeostasis (GO:0042592, *n* = 15, FDR = 9.17 × 10^− 5^), complement activation (GO:0006956, *n* = 8, FDR = 2.04 × 10^− 9^), acute inflammatory response (GO:0002526, *n* = 10, FDR = 2.34 × 10^–11^), humoral immune response (GO:0006959, *n* = 13, FDR = 8.42 × 10^–11^), etc., according to the String DB (version 12.0)^29^. In the moderate (*n* = 9) and low (*n* = 4) abundance transition zones, mutual identifications were scarce, with most identifications coming from PowerNovo, and included proteins involved in cellular localization. The low-abundance zone is shown with limitations, some of the proteins lack quantitative annotation but it should be noticed that the use of both strategies expanded the identification zones.

Seventy proteins were common for both PLGS and PowerNovo strategies (Fig. 2a) and associated with secreted and predominantly localized in the extracellular space (GO:0005615, *n* = 33, FDR = 4.73 × 10^–35^), blood microparticle (GO:0072562, *n* = 28, FDR = 3.75 × 10^–50^), secretory granule (GO:0030141, *n* = 17, FDR = 6.60 × 10^–13^) and others. Based on molecular function, the proteins can be classified as acute inflammatory response (GO: 0002526, *n* = 8, FDR = 1.69 × 10^− 8^), humoral immune response (GO: 0006959, *n* = 9, FDR = 1.43 × 10^− 6^), response to stress (GO: 0006950, *n* = 23, FDR = 1.05 × 10^− 6^). The common identifications set is proteins involved in the acute-phase response (GO:0006953, *n* = 6, FDR = 3.68 × 10^− 7^), acute inflammatory response (GO:0002526, *n* = 8, FDR = 1.69 × 10^− 8^), negative regulation of hydrolase activity (GO:0051346, *n* = 12, FDR = 1.69 × 10^− 8^), positive regulation of phagocytosis (GO:0050766, *n* = 5, FDR = 0.00014), negative regulation of very-low-density lipoprotein particle remodelling (GO:0010903, *n* = 3, FDR = 8.78 × 10^− 5^), humoral immune response (GO:0006959, *n* = 9, FDR = 1.43 × 10^− 6^), and inflammatory response (GO:0006954, *n* = 11, FDR = 2.56 × 10^− 6^).

We compared the in-depth analysis of our DIA dataset with available datasets: “Identification of Plasma Biomarkers from Rheumatoid Arthritis Patients Using an Optimized SWATH Proteomics Workflow” (PXD045171, TripleTOF 6600^30^ and “Quantitative variability of 342 plasma proteins in a human twin population” (PXD001064, TripleTOF 5600^31^. Our results are comparable with at least PXD001064 dataset with in-depth of protein concentration nearly 10 fg/mL (see Supplementary Materials, Figures S1 and S2).

### Features of plasma protein composition in comparison groups

Table 2 lists proteins (*n* = 8) distinguishing the “High” group from others. This group of proteins is involved in immune response, lipid metabolism, and oxygen/iron transport and all the listed proteins are secretory with a status “evidence at protein level” as in the UniProtKB, detected in plasma (Protein Atlas), and have at least one identified proteotypic peptide (PTP). Calculated concentrations in athletes generally correspond to the reported normal ranges (column “Normal range”) and controls though these ranges are not still standardized in clinical diagnostics.

**Table 2:** Features of the protein composition in the “High” group samples relative to other comparison groups.

UniProt ID	Name	Mw, kDa	Lengh	Gene	Localization	Biological processes	Fold changes (*p*-value)			Sequence coverage, %		Concentration, mg/L				
							High vs. Low	High vs. Moderate	High vs. all	PLGS	PowerNovo	High	Moderate	Low	Control	Normal range
Male																
P05090	Apolipoprotein D	21	189	*APOD*	Extracellular space	Transport	–	1.71 (*p* = 0.078)	–	29	–	98 ± 32 (*n* = 6)	55 ± 11 (*n* = 6)	– (*n* = 1)	83 ± 39 (*n* = 10)	100–120 [32]
P02774	Vitamin D-binding protein	53	474	*GC*	Extracellular space	vitamin D metabolic process	0,9 (*p* = 0.84)	1.1 (*p* = 0.74)	1.1 (*p* = 0.74)	39	3	286 ± 84 (*n* = 19)	268 ± 94 (*n* = 34)	301 ± 104 (*n* = 5)	217 ± 11 (*n* = 10)	200–600 [33] 176–623 [34]
P00739	Haptoglobin-related protein	39	348	*HPTR*	Extracellular space	Hemoglobin binding	1.6 (*p* = 0.029)	1.4 (*p* = 0.047)	1.4 (*p* = 0.0209)	38	12	361 ± 94 (*n* = 18)	326 ± 226 (*n* = 36)	259 ± 113 (*n* = 11)	318 ± 245 (*n* = 8)	–
P04433	Immunoglobulin kappa variable 3–11	13	115	*IGKV3-11*	Extracellular space	Immune response	0.82 (*p* = 0.031)	1.11 (*p* = 0.84)	1.07 (*p* = 0.42)	77	16	64 ± 38 (*n* = 17)	72 ± 42 (*n* = 37)	113 ± 66 (*n* = 9)	148 ± 202 (*n* = 10)	–
P02749	Beta-2-glycoprotein 1	32	345	*APOH*	Extracellular space	Triglyceride metabolic process	1.52 (*p* = 0.055)	1.28 (*p* = 0.024)	1.3 (*p* = 0.0136)	55	–	297 ± 88 (*n* = 15)	246 ± 103 (*n* = 27)	225 ± 93 (*n* = 6)	143 ± 79 (*n* = 10)	200 [35]

Lengh, aa – protein length in amino acid residues; Localization – localization in the cell; Mw – molecular weight; PLGS – Protein Lynx Global Server; Normal range – the range of protein concentration in plasma for a healthy person (literature data, confirmed by a reference); Concentration, mg/L – assessment for groups of athletes based on PLGS data; p-values calculated using the Mann-Whitney U test with the Benjamini-Hochberg correction.

The combination of PLGS library search and PowerNovo *de novo* sequencing provided complementary data, increasing overall reliability. In some cases (Table 3), these two algorithms successfully complemented each other, increasing protein sequence coverage. A full comparative analysis is available in Supplementary Table S1.

**Table 3:** Increasing the protein sequence coverage using the de novo protein sequencing strategy.

Gene	Name	Samples		Seq coverage AA (%)		PowerNovo AA coverage increase
		PLGS	Power Novo	PLGS	Power Novo	
Male						
ALB	Albumin	74	74	570(94)	359(59)	10
APOA1	Apolipoprotein A1	74	66	237 (89)	175 (66)	8
PZP	Pregnancy zone protein	48	16	573 (39)	106 (7)	61
HBA1	Hemoglobin subunit alpha	37	10	132 (93)	87 (61)	9
HBD	Hemoglobin subunit delta	34	9	91 (62)	54 (37)	13
SERPINA2	Alpha-1-antitrypsin-related protein	17	8	80 (19)	23 (5)	8
IGHV3-48	Immunoglobulin heavy variable 3–48	27	9	31 (26)	20 (17)	20
Female						
PZP	Pregnancy zone protein	16	4	457 (31)	85 (6)	23
HBD	Hemoglobin subunit delta	11	4	115 (78)	62 (42)	13
IGLC7	Immunoglobulin lambda constant 7	19	4	34 (32)	24 (23)	9
FGA	Fibrinogen alpha chain	19	4	403 (47)	65 (8)	9
IGHG2	Immunoglobulin heavy constant gamma 2	19	9	286 (72)	53 (13)	7

Seq coverage AA (%) – length of identified peptides and the coverage (%) of protein sequence identifications when performing search using the PLGS or PowerNovo approach. The list of sequences is provided in Supplementary Table S1.

**Fig. 4:**
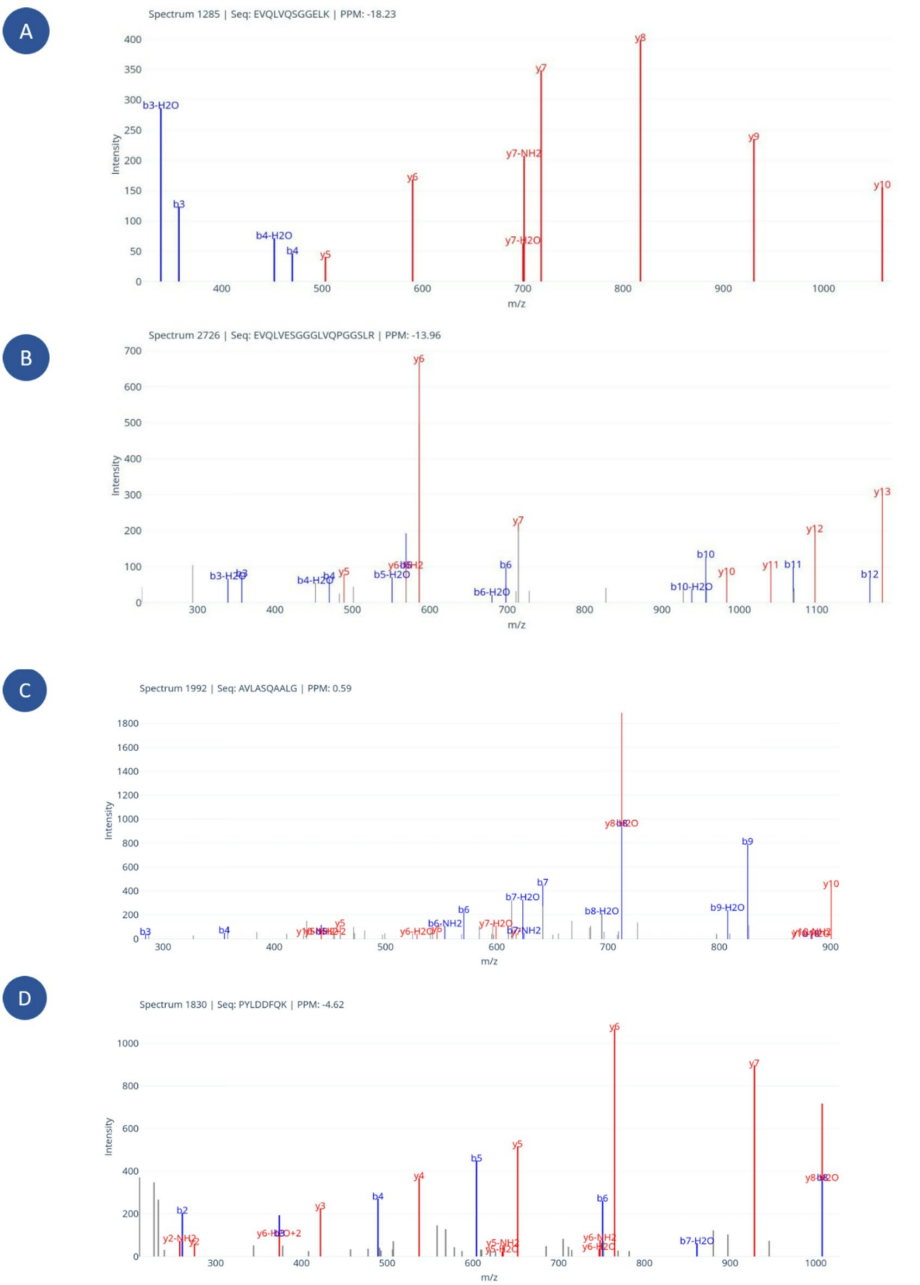
Examples of identifications showing the match between theoretical ions (calculated from the predicted peptide) and the spectrum. The figures demonstrate peptides identified exclusively by PowerNovo and not by PLGS. (**A**, **B**) peptides of IGHV3-48; (**C**) peptide of albumin and (**D**) apolipoprotein A1. *b-* and *y-*ions are marked in blue and red for spectra after PowerNovo identification. Ions b and y are matched with tolerance 0.05 m/z including ions with water and ammonia losses using Python/Peptacular package.

Figure 4 shows examples of identifications where PowerNovo succeeded compared to PLGS.

### Clinical data

**Fig. 5:**
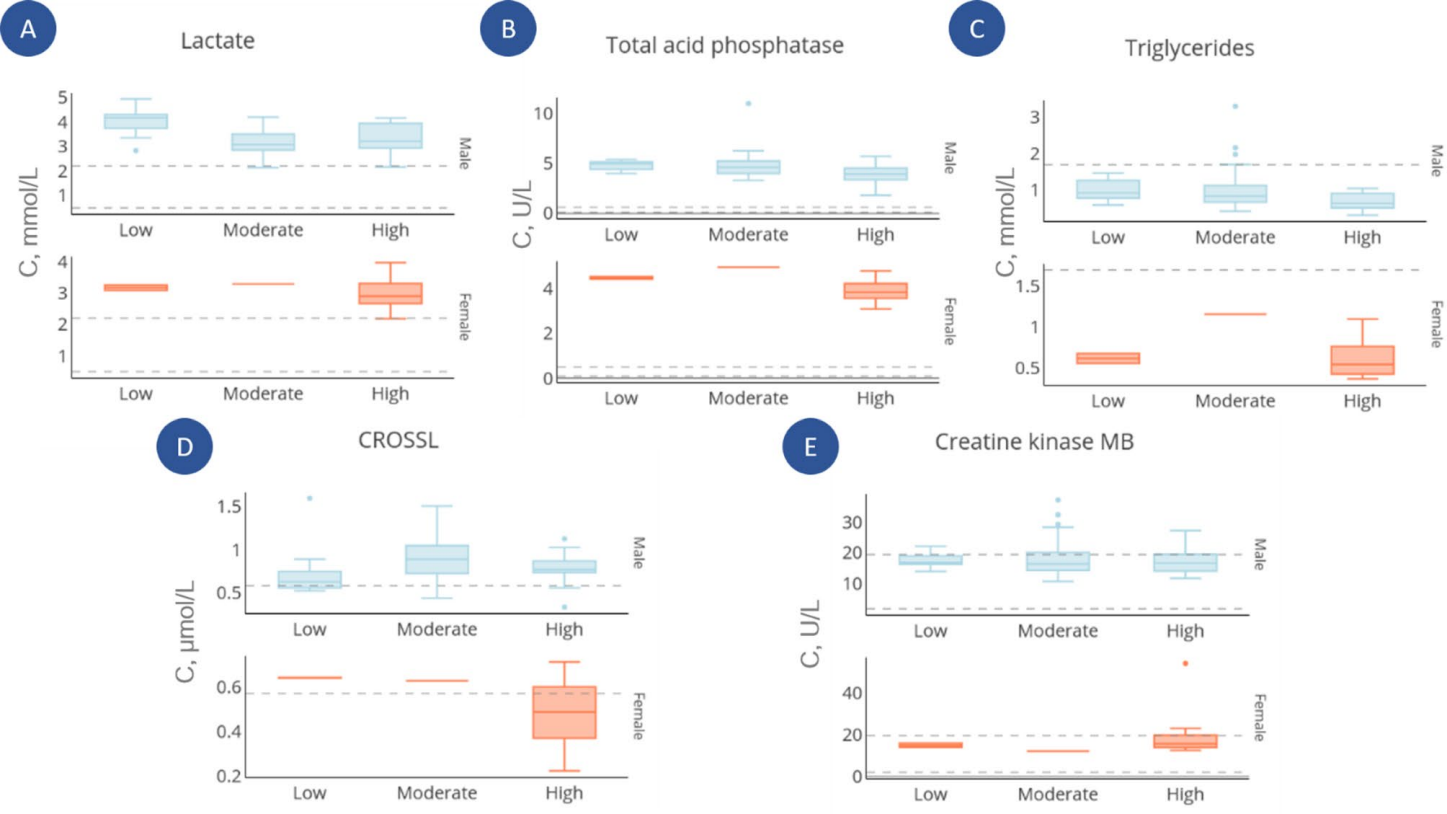
Box plots for six clinical blood parameters differing between the “High” group and other groups. Parameters measured in males and females are shown in blue and red, respectively: (**a**) lactate; (**b**) total acid phosphatase; (**c**) triglycerides; (**d**) CROSSL – C-terminal telopeptides of type I collagen; (e) creatine kinase. Dashed lines show reference values of normal range for every clinical parameter. If minimal reference value is equal to zero, no lower line is shown on the plot.

We analyzed changes in biochemical parameters among the three groups and discovered modulation of individual biochemical parameters (Fig. 5).

Most measured biochemical parameters were within or near the upper limit of the normal range. Exceedances were observed for CROSSL (a marker of bone resorption), lactate, and total acid phosphatase, and these parameters showed statistically significant differences between the groups of men. Specifically, blood lactate levels in men were approximately 30% higher in the Low group compared to the High (*p* = 0.0105) and Moderate (*p* = 0.00058) groups. Markers of lipid metabolism were reduced in the high- and moderate-activity groups relative to the Low group: total acid phosphatase decreased by 25% (*p* = 0.0085) and triglycerides by 45% (*p* = 0.0098). Conversely, CROSSL levels increased by 40% in the Moderate group versus the Low group (*p* = 0.0408) and by 20% in the High group versus the Low group (*p* = 0.059). Creatine kinase activity was elevated by 70% in the High group (*p* = 0.0448) and by 20% in the Moderate group (*p* = 0.0448) compared to the Low group. Similar trends were observed in female samples, although due to the smaller sample size, these findings should be interpreted with caution and considered preliminary.

## Discussion

Advances in omics technologies and the availability of mass spectrometry support a new direction in sports medicine such as finding biomolecular indicators of competitive activity and athlete health. Maintaining health, detecting negative post-competition effects, and optimizing diet, load, and recovery are crucial for professional athletes^32,33^. We note that long-term professional sports (for several years to decades) may be associated with modulation of metabolic processes and, thus, to changes in the profile of circulating blood proteins^21^. Prolonged engagement in professional sports is associated with morphofunctional changes (increased muscle/capillary density, heart volume)^34–36^, metabolic adaptations (improved energy metabolism, mitochondrial function)^37–39^, and hormonal/immune alterations^13,40–42^. Investigating the relationships between sport, load intensity, and adaptive changes is a key aspect of improving training, minimizing negative effects, and optimizing nutrition^43,44^.

In this study, the analysis of the plasma proteome from elite athletes was performed using two different approaches of mass spectrometry data processing: PLGS is based on a sequence library search, and PowerNovo is designed for de novo peptide sequencing that does not employ genetic databases or spectral libraries. When analyzing complex samples, identification results can vary significantly depending on the search algorithm used. Previously, researches of Shteynberg (2013)^45^, Ivanov (2015)^46^, and Audain (2017)^47^ demonstrate that proteomic search engines show discrepancies both in the number of proteins detected and in their composition.

To date, approaches based on de novo sequencing are more prone to redundant identifications compared to the library-based solutions, although the latter are not impeccable. The main reason for redundancy is the inherently low accuracy of peptide sequence prediction when working with mass spectrometry data^48^. This issue is caused by both the imperfection of the algorithms themselves, the completeness and quality of the training datasets, and the inevitable shortcomings of mass spectrometric analysis likewise the presence of noise, the absence of fragment ion peaks, ion current failures, and other factors^48–50^. All this leads to the appearance of potentially false identifications.

The problem is aggravated by the methodological limitations of the de novo approaches themselves. Many algorithms generate only short peptide tags (three or even two amino acid residues long) increasing the risk of incorrect identifications^50^. Furthermore, for de novo sequencing, a standardized method for estimating the false discovery rate (FDR), similar to that used in traditional database searching, has not yet been developed. Consequently, a predicted peptide sequence may contain errors even with a high individual score^51^. This creates a situation where the primary data requires subsequent rigorous filtering and confirmation using hybrid approaches^52^.

In this study, to minimize these effects, stringent filtering criteria and additional validation using PLGS were applied. It is important to emphasize that the use of two approaches in modern proteomics is aimed mainly not for increasing the number of identifications, but for the mutual confirmation of results and increasing the reliability of the findings. Pre-analytical, analytical, and post-analytical procedures for the sports groups were performed under identical conditions to mitigate batch effects.

**Fig. 6:**
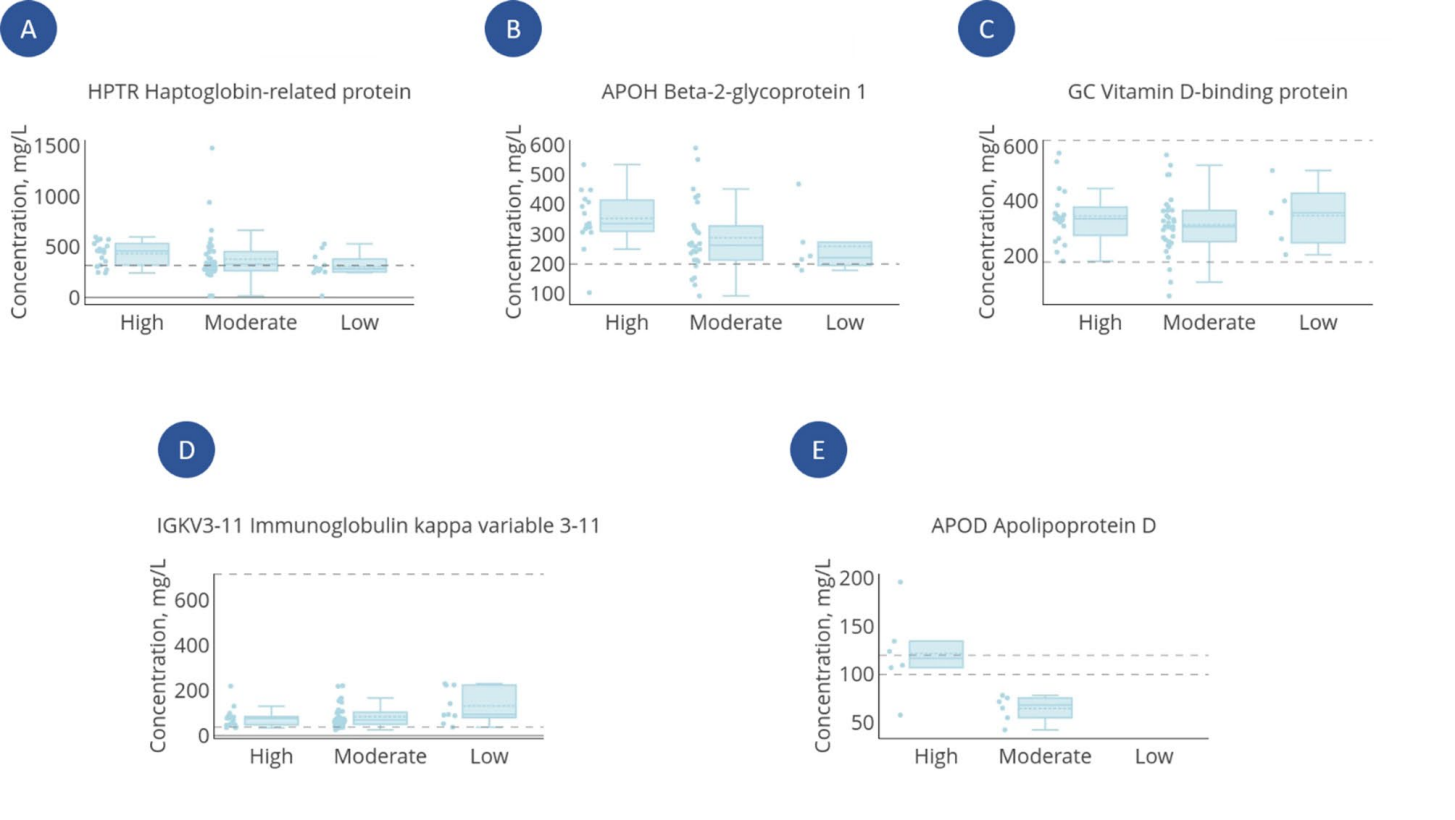
Box plots for DEP between the “High” group and other groups. Parameters measured in males: (**a**) plasma haptoglobin-related protein; (**b**) beta-2-glycoprotein I; (**c**) vitamin D-binding protein; (**d**) immunoglobulin kappa variable 3–11; (**e**) apolipoprotein D. Dashed lines show reference values of normal range or control group.

We observed variations in protein content among the three groups, specifically in the modulation of acute-phase proteins involved in immune response, complement activation, coagulation, and cholesterol metabolism (Fig. 6). In the “High” vs. “Low” comparison, plasma haptoglobin-related protein (HPR) increased (FC 1.6, *p* = 0.029, Fig. 6a). HPR is a glycoprotein that binds hemoglobin effectively, is 90% homologous to haptoglobin (Hp)^53^, and plays a key role in tissue protection and preventing oxidative damage^54^. Its role in sports medicine is poorly understood, though it is considered a potential modulator of inflammation^54^. Normal HPR values are not standardized but correlate with Hp levels. Little is known about HPR levels in healthy people but our data are in good agreement with the protein content in the control dataset and athlete groups. An increase is also seen in “High” vs. “Moderate” (FC = 1.4, *p* = 0.047, Fig. 6a). Measurements of hematological parameters like hemoglobin, hematocrit, and albumin, might be important in the analysis of individual shifts in circulating plasma volume related to the athlete’s hydration status at the time of sample collection^55,56^. Our study revealed no statistically significant differences in albumin levels between the comparison groups, with albumin concentrations showing no variation in either DIA-MS measurements or biochemical analysis.

Beta-2-glycoprotein I (APOH) is one of the few proteins regulating the complement system and coagulation. The protein consists of five domains and is primarily synthesized in the liver and secreted into the bloodstream. It binds phospholipids, clears apoptotic cells, and is a cofactor for antiphospholipid antibodies^57,58^. Elevated levels of APOH and anti-APOH antibodies are risk markers for thrombosis (e.g., in antiphospholipid syndrome)^59^. Elevated APOH levels in such patients may reflect the activation of vascular-immune mechanisms associated with tissue repair. Normally, APOH circulates in plasma at a concentration of around 200 mg/L^60^. In our study, plasma levels correspond to literature and control data. The “High” group showed higher APOH levels vs. “Low” (FC = 1.52, *p* = 0.055) and “Moderate” (FC = 1.28, *p* = 0.024, Fig. 6b).

The vitamin D-binding protein (GC) binds vitamin D and its metabolites, and transports them through the bloodstream. It is a key factor in maintaining vitamin D levels and determines how efficiently the body utilizes it (Fig. 6c). In addition, GC also binds to actin, potentially involved in immune responses^61^, modulates macrophage activation, and binds fatty acids, indicating its role in the regulation of lipid metabolism^61^. This may influence both inflammatory responses and fat metabolism. The plasma concentration of GC is ≥ 176 mg/L^62^.

Studying molecular factors in oxidative stress regulation is important in sports medicine^16^. Different types and intensities of training lead to variations in oxidative stress status among athletes^16,63^. In our study, apolipoprotein D (ApoD) was predominantly detected in the “High” and “Moderate” groups (FC = 1.7, *p* = 0.078, Fig. 6e). The ApoD is expressed in various organs and tissues such as the liver, intestine, pancreas, kidney, spleen, and the central nervous system^64^. The protein is secreted into the bloodstream, where its normal concentration is 100–120 mg/L^65^. Increased ApoD is associated with reduced oxidative stress and inflammation, possibly by binding arachidonic acid and altering its pro-inflammatory metabolism^56^. This omega-6 polyunsaturated fatty acid can be metabolized to produce a wide range of pro-inflammatory mediators. ApoD acts as a sequestrant, blocking and/or altering the metabolism of arachidonic acid^66^. Thence, ApoD activation may indicate a compensatory oxidative stress regulation mechanism^66^. We revealed significant differences between the male comparison groups for immunoglobulin kappa variable 3–11 (Fig. 6d). However, this information requires further study.

Despite the number of women included in the study constrains the statistical significance, three proteins associated with immune processes were nevertheless identified: alpha-1B-glycoprotein (A1BG), fibrinogen alpha chain (FGA) and immunoglobulin heavy variable 3–7 (IGHV3-7)^67,68^. The study results showed that the “High” group showed higher A1BG levels vs. “Low” (FC = 1.6) and “Moderate” (FC = 1.4), higher FGA levels vs. “Low” (FC = 1.5) and “Moderate” (FC = 1.3), and higher IGHV3-7 levels vs. “Low” (FC = 7). These data require further development and validation. However, they fit into the biological understanding of the processes.

Biochemical parameter measurements also support segmentation within comparison groups. A prior population study (> 3,600 athletes) showed that myoglobin, uric acid, creatine kinase, and ALT levels^32^ are important for segmenting athlete phenotype into “catabolism” and “anabolism” during recovery, consistent with our results. We observed a 46% decrease in triglycerides (“High” vs. “Low”, *p* = 0.0098) and a decrease in lactate (*p* = 0.01), consistent with literature^69^. As expected, creatine kinase increased by 65% in the “High” group (*p* = 0.0448)^70^, consistent with DIA-MS results (Table 2). Further research can be scaled by both sport disciplines and participant numbers.

## Conclusion

This pilot study is focused on analyzing the fundamental feasibility of using tandem mass-spectrometry proteomic data to identify features of the protein composition in the blood of elite athletes engaged in sports with varying exercise intensity. The study proposed two approaches for proteomic data analysis: protein search and identification using a protein sequence database (PLGS) and de novo sequencing of peptide and protein sequences (PowerNovo). The combination of these approaches makes it possible to increase the reliability of identification and, to a certain extent, to increase the coverage of protein sequences by peptides. Both approaches allowed the identification of 80 proteins, that are secreted and involved in transport, immune response, and inflammation. Proteins ApoD and GC with group-specific characteristics were identified, as well as proteins that were differentially expressed between the “High” and “Low” groups, including acute phase and inflammation proteins (HPR, IGKV).

Among the biochemical and haematological changes, the decrease in the levels of lactate and lipid metabolism markers (total acid phosphatase and triglycerides) is severely emphasized, which indicates adaptation to physical activity and an increase in oxidative metabolism and lipolysis.

The work has a bidirectional significance: from a methodological point of view, it demonstrates the possibility of integrating database searching and de novo sequencing to increase the reliability and depth of proteomic profiling; from a biological perspective, the obtained data contribute to the development of ‘sportomics’ by identifying protein and clinical patterns associated with adaptation to physical exertion. The study not only confirms previously described patterns but also proposes promising complex markers for the potential monitoring of the training process and prevention of pathological conditions. The work illustrates how modern multiplex technologies enable a transition from the analysis of single molecules to an integrative assessment, increasing the efficiency and informativeness of research in sports physiology.

## Limitations of the study

This study has limitations: the number of participants and sports was limited. This pilot study exhibits a gender imbalance; thus, results concerning women should be interpreted with caution. Future study designs should account for numerous factors including individual diet, hydration, hormonal/circadian rhythms, sleep quality, menstrual cycle phase (women), and psycho-emotional state. Smoking is also a potential influencing factor. These factors may affect plasma protein levels.

The main objective of the work was a comparative analysis between the groups of athletes, performed under identical conditions, whereas the quantitative data from the control group (obtained previously) and the literature normal ranges are provided solely to assess the expected range of protein concentrations and were not used in statistical tests.

It is important to provide an explanation for the moderate number of protein identifications in our study, which is due to a combination of methodological factors, including the features of sample preparation (without fractionation), the choice of analytical platform (micro-flow in chromatographic separation), and stringent data processing criteria (requirements for the measurement accuracy of precursor and fragments, frequency of occurrence in samples).

Scaling up the study is important. Future work will help overcome these limitations.

## Supplementary Information

Below is the link to the electronic supplementary material.

Supplementary Material 1

Supplementary Material 2

## Data Availability

The datasets generated and analysed during the current study are available in the MassIVE repository, full member of the ProteomeXchange consortium – https://massive.ucsd.edu/ProteoSAFe/QueryPXD?id=PXD067642.

## Abbreviations

MaxO2: Maximal oxygen uptake
PLGS: Protein Lynx Global Server
MS: Mass spectrometry
CROSSL: C-Terminal telopeptides of type I collagen
A2M: Alpha-2-macroglobulin
APOH: Apolipoprotein H (Beta-2-glycoprotein I)
ApoD: Apolipoprotein D
ALT: Alanine aminotransferase
