# Supplementary material for: Changes in the proteomic profile of athletes’ plasma associated with exercise intensity
Source: Sci Rep. 2026 Mar 19;16:14205. doi: 10.1038/s41598-026-44729-5 (PMC13139415; doi:10.1038/s41598-026-44729-5)
Supplement: Supplementary file 2 — Supplementary Material 2 [file 41598_2026_44729_MOESM2_ESM.docx]

**Supplementary Materials**


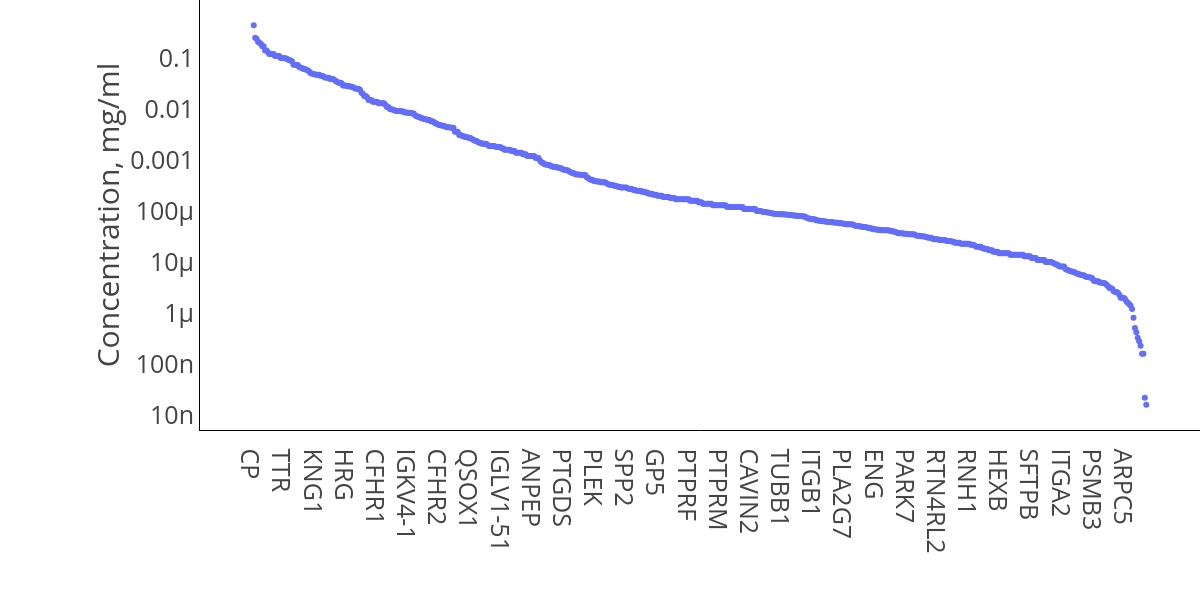


**Figure S1.** Concentrations of identified circulating proteins for the dataset: “Identification of Plasma Biomarkers from Rheumatoid Arthritis Patients Using an Optimized SWATH Proteomics Workflow” (PXD045171). Data are presented for a group of healthy volunteers (n = 20). A total of 663 proteins were identified using an optimized SWATH-based proteomics approach on the TripleTOF 6600 platform. A distinctive feature of the project was the identification of potential proteoforms^1^. No FDR threshold was applied.

Plasma proteins shown on the left may be assigned to the range of high-copy number proteins (>1 μg/mL); proteins grouped in the center correspond to the range of moderate-copy number proteins (1 μg/mL to 10 ng/mL); proteins shown on the right correspond to the range of low-copy number proteins (<10 ng/mL). Genes of identified proteins are shown on the X-axis.


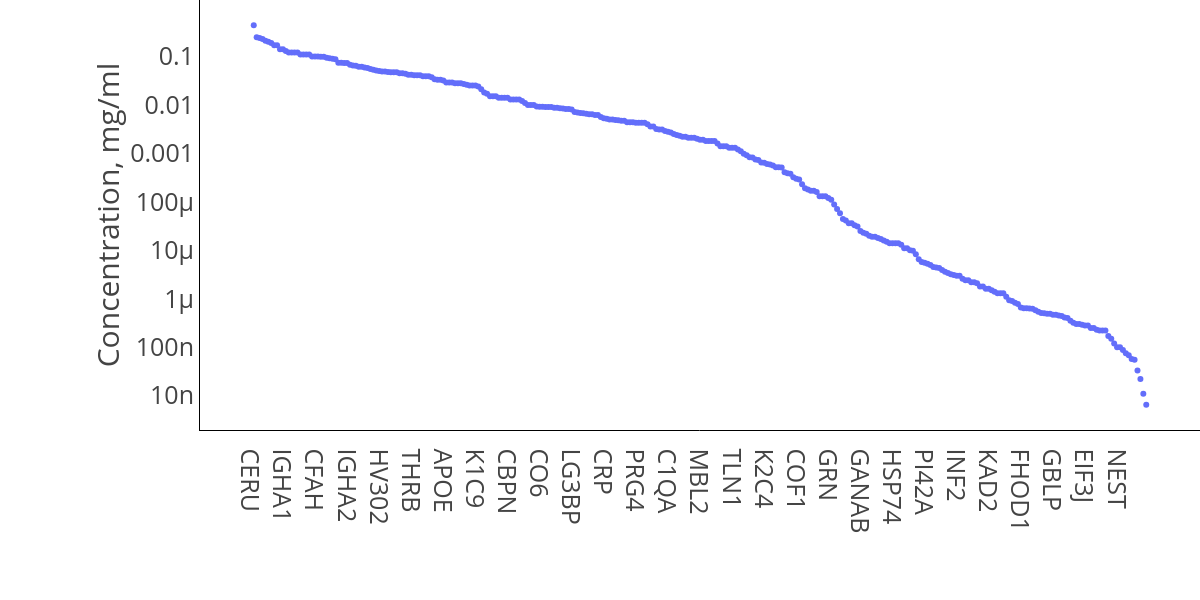


**Figure S2.** Concentrations of identified circulating plasma proteins for the dataset: “Quantitative variability of 342 plasma proteins in a human twin population” (PXD001064). Data are presented for a group of healthy human twins (n = 232). Quantitative variation was detected for 342 proteins using DIA analysis on the TripleTOF 5600 platform^2^. The parent mass tolerance was set to 50 ppm, and the monoisotopic fragment mass tolerance to 0.5 Da. An FDR of 10% was applied.

Plasma proteins shown on the left may be assigned to the range of high-copy number proteins (>1 μg/mL); proteins grouped in the center correspond to the range of moderate-copy number proteins (1 μg/mL to 10 ng/mL); proteins shown on the right correspond to the range of low-copy number proteins (<10 ng/mL). Genes of identified proteins are shown on the X-axis.

Literature

(1) Jin, L.; Wang, F.; Wang, X.; Harvey, B. P.; Bi, Y.; Hu, C.; Cui, B.; Darcy, A. T.; Maull, J. W.; Phillips, B. R.; Kim, Y.; Jenkins, G. J.; Sornasse, T. R.; Tian, Y. Identification of Plasma Biomarkers from Rheumatoid Arthritis Patients Using an Optimized Sequential Window Acquisition of All THeoretical Mass Spectra (SWATH) Proteomics Workflow. Proteomes 2023, 11 (4), 32. https://doi.org/10.3390/proteomes11040032.

(2) Liu, Y.; Buil, A.; Collins, B. C.; Gillet, L. C. J.; Blum, L. C.; Cheng, L.-Y.; Vitek, O.; Mouritsen, J.; Lachance, G.; Spector, T. D.; Dermitzakis, E. T.; Aebersold, R. Quantitative Variability of 342 Plasma Proteins in a Human Twin Population. Mol. Syst. Biol. 2015, 11 (1), 786. https://doi.org/10.15252/msb.20145728.
